# Remarks on Electricity in Medicine and Surgery

**Published:** 1903-02

**Authors:** Joseph C. Clark

**Affiliations:** Olean, N. Y., 119 Laurens Street.


### Remarks on Electricity in Medicine and Surgery.

By JOSEPH C. CLARK, M. D., Olean, N. Y.

IN THESE times of rapid development of new electrical apparatus and methods, a medical man not equipped with a good dry cell or direct current, and an up-to-date faradic battery must be considered decidedly unprepared to deal with many conditions of ill-health. The well-known power of these currents to relieve pain in nerves and to loosen the tendon attached by infiltrations to its sheath, and thus restore crippled function still holds good, and will continue to do so as long as these currents are known. Some become more ambitious and enter a field of electricity that is full of battle, unrest and wide diverging opinions as to its ability to do half that is claimed, and that is the field of the mysterious and seductive static electricity and its more mysterious production of *x*-ray.

A few years ago a 4 or 6 revolving plate static machine was considered all that one could wish. Then the manufacturers began to talk static breeze and the apparatus took on the formidable size of 16 to 24 revolving plates, and improvements in insulation, ball bearings and charges rapidly took place. A reaction has now set in, and an apparatus with 10 revolving plates will do all that is required with much less danger of unpleasant effects upon the patient.

In using the static electricity during the past eight years, I am very favorably impressed with its good effects in tertiary syphilis, such as prompt relief to nodes upon the skin, glandular enlargements and ulcers and the very rapid disappearance of lesions, in connection with specific treatment. A patient recently came under my care who had a sinus following an operation for appendicitis of eighteen months' duration, and who had been taking specific treatment for months. He has shown marked improvement under the use of the static breeze and *x*-ray exposures combined with specific treatment. I consider the static breeze and *x*-ray treatment as favorable to relieve syphilitic disease as alcohol is unfavorable to its cure.

The tonic effect of static electricity does not exceed that of the galvanic or faradic; the careful and thorough use of the galvanic and faradic constitutes one of the greatest tonics and stimulants that can be brought to bear upon the human system. It awakens torpid conductivity of every nerve thread in the body and its greatest benefits can be demonstrated in the aged in whom loss of superficial sensation can be proved, only to quickly return after a few treatments by the galvanic and faradic currents. The galvanic should be used never longer than fifteen minutes to begin with, for fear of reaction, such as chills and nervousness. But it is some trouble to disrobe, and the exposure often deters the medical man from using these currents. The static apparatus here comes in as a quick and convenient method of giving electricity and, as an enthusiastic patient once remarked, "It is good for whatever ails you."

It is as curative as any medicine known for rheumatism; besides, medical treatment can be employed with it. The static enthusiasts remind one of those mildly deranged and superior people called christian scientists: it's static or nothing.

I might furnish a long list of diseases favorably affected by static electricity, but will only say that as a means of watching a patient, and especially in many chronic diseases, there is no method known that gives one as ample opportunity for observation and benefit.

The *x*-ray will photograph the human skeleton, but I have known men to fail in securing a skiagraph, and one physician denied that it would produce a picture. That the *x*-ray will cure the superficial forms of lupus, scarcely admits of a doubt, yet this is denied daily by many eminent men. Shall we put them in the class with the man who failed to secure the skiagraph?

The skin specialist with his arsenic and methyls and other stomach disturbers, cannot, in my opinion, show the results obtained by *x*-ray treatment in a large number of skin troubles and, as already remarked, there is no reason why internal medication should not be used in connection with the *x*-ray, yet as a matter of fact it is often unnecessary. The use of the $x$-ray in superficial epithelioma is fairly satisfactory. Some of the small and very chronic cases are quickly cured. The more acute the condition the surer the failure to relieve.

Carcinomatous conditions are relieved of pain and discharge, lessens under its use. This is more marked where the process is very slow and chronic. The $x$-ray will not cure these cases, but the use of the $x$-ray after the operation upon enlarged glands and returning nodules seems fairly successful, and it is to be hoped the $x$-ray will be sufficient to render the extreme dissection under the pectoral muscles unnecessary.

The teaching of electro-therapeutics in our colleges, and its employment in our large hospitals will increase as the demand for more knowledge of this great aid to the practice of medicine and surgery becomes apparent.

119 LAURENS STREET.

